# Harnessing the Heart’s Magnetic Field for Advanced Diagnostic Techniques

**DOI:** 10.3390/s24186017

**Published:** 2024-09-18

**Authors:** Tarek Elfouly, Ali Alouani

**Affiliations:** Department of Electrical and Computer Engineering, Tennessee Technological University, Cookeville, TN 38505, USA; aalouani@tntech.edu

**Keywords:** magnetocardiography (MCG), electrocardiography (EEG), superconducting quantum interference devices (SQUIDs)

## Abstract

Heart diseases remain one of the leading causes of morbidity and mortality worldwide, necessitating innovative diagnostic methods for early detection and intervention. An electrocardiogram (ECG) is a well-known technique for the preliminary diagnosis of heart conditions. However, it can not be used for continuous monitoring due to skin irritation. It is well known that every body organ generates a magnetic field, and the heart generates peak amplitudes of about 10 to 100 pT (measured at a distance of about 3 cm above the chest). This poses challenges to capturing such signals. This paper reviews the different techniques used to capture the heart’s magnetic signals along with their limitations. In addition, this paper provides a comprehensive review of the different approaches that use the heart-generated magnetic field to diagnose several heart diseases. This research reveals two aspects. First, as a noninvasive tool, the use of the heart’s magnetic field signal can lead to more sensitive advanced heart disease diagnosis tools, especially when continuous monitoring is possible and affordable. Second, its current use is limited due to the lack of accurate, affordable, and portable sensing technology.

## 1. Introduction

**Figure 1:**
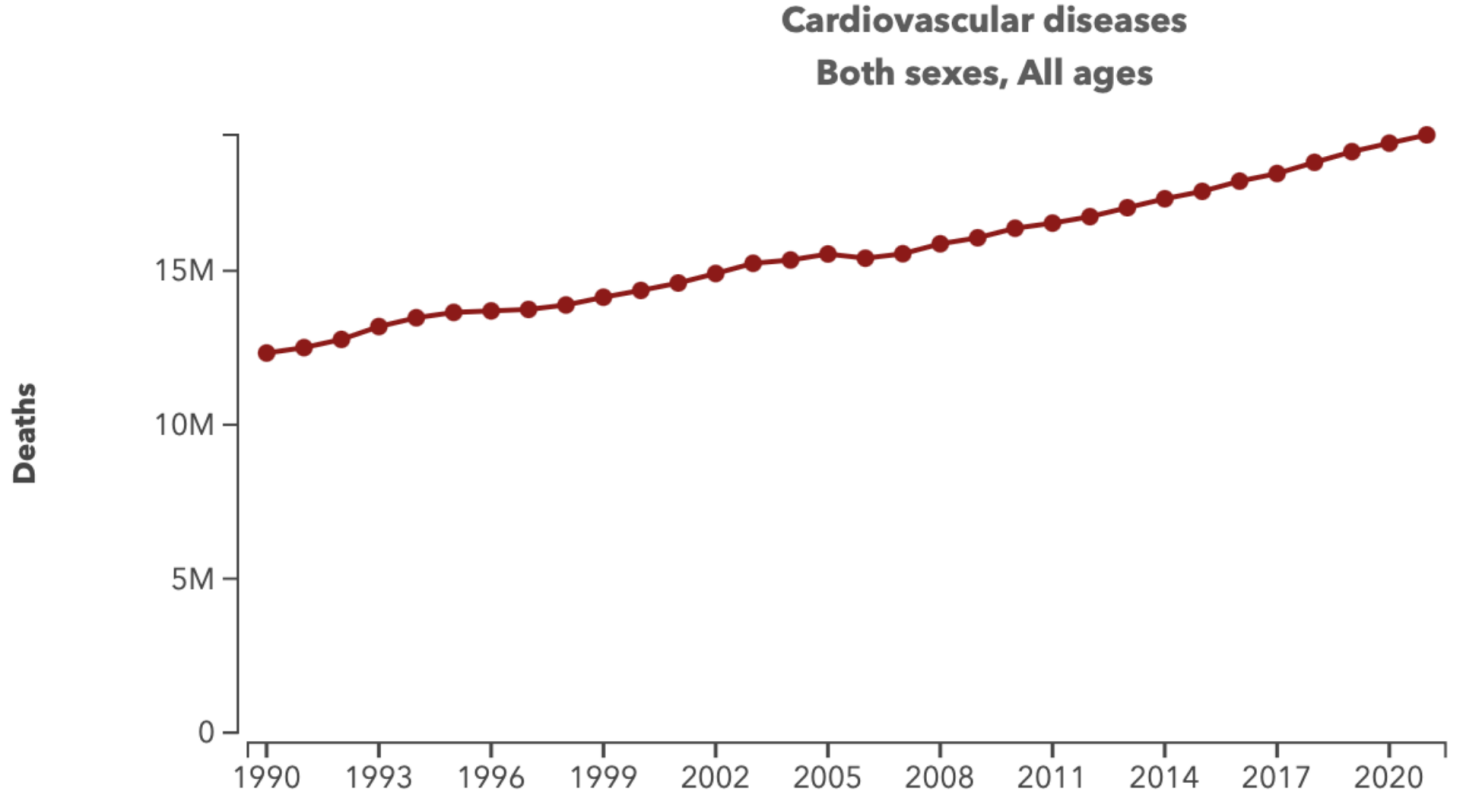
Number of deaths due to cardiovascular diseases, 1990–2021. Source: Institute for Health Metrics and Evaluation (IHME). GBD Compare Data Visualization. Seattle, WA: IHME, University of Washington, 2020. Available online: https://vizhub.healthdata.org/gbd-compare/ (accessed on 18 May 2024).

Cardiovascular diseases (CVDs) impact the heart and blood vessels, resulting from a mix of socio-economic, metabolic, behavioral, and environmental risk factors [1]. These factors include high blood pressure, unhealthy diet, high cholesterol, diabetes, air pollution, obesity, tobacco use, kidney disease, physical inactivity, excessive alcohol consumption, and stress [1]. For decades, CVDs have been the leading cause of death worldwide with a significant increase from 12.1 million deaths in 1990 to 17.9 million deaths in 2021 (Figure 1), and 85% of these deaths are due to ischemic heart disease [2,3]. This number represents one-third the number of deaths worldwide [2,4]. It is worth mentioning that the world population also increased during the same duration from 5.3 to nearly 8 billion. This means that with all the advancement in the medical field, the number of deaths due to CVDs did not decrease and that more cardiological research is needed. CVDs encompass various disorders of the heart and blood vessels, including coronary heart disease, cerebrovascular disease, rheumatic heart disease, and other related conditions [5]. Diagnosing heart diseases is critically important for early detection, prevention, and treatment planning. Identifying heart disease at an early stage allows for timely intervention to manage risk factors and prevent disease progression. Early diagnosis helps prevent serious complications, such as heart attacks, strokes, and heart failure. An accurate diagnosis enables the development of a tailored treatment plan, including medications, lifestyle changes, and potential surgical interventions. It also allows for continuous monitoring and adjustment of treatment to ensure optimal outcomes. Early detection and management can reduce the need for expensive emergency care, hospitalizations, and advanced treatments, thus lowering overall healthcare costs. An ECG is the only known affordable technique to perform preliminary heart diagnosis. Unfortunately, an ECG lacks the sensitivity necessary for accurate diagnosis [6,7]. Furthermore, an ECG can not be used for continuous monitoring due to the skin irritations. Thus, finding new techniques to diagnose heart diseases is essential for enhancing the accuracy, early detection, and overall effectiveness of heart disease management.

Diagnosing heart diseases has evolved from using a stethoscope [8] to an ECG [9,10]. An ECG is used to record the electrical activity of the heart [11]. An ECG is a fundamental, noninvasive, and cost-effective diagnostic tool widely used to assess heart health. It offers immediate results and is effective for monitoring cardiac conditions. However, it has limitations [12] in detecting structural heart issues and capturing intermittent events and requires skilled interpretation to avoid misdiagnosis. Additional tests may be necessary to complement ECG findings and provide a comprehensive cardiac assessment due to its sensitivity to external factors [6]. Patients also could suffer from an allergy due to the electrodes placed on their skin [13]. The Holter monitor is a portable ECG device worn for 24–48 h to record heart activity over time to detect irregularities [14]. The Holter monitor is a valuable tool for diagnosing and managing cardiac arrhythmias, providing continuous monitoring that captures transient events and correlates them with patient-reported symptoms. However, it has limitations, including the potential for missed events due to the short monitoring period, the need for patient compliance, and susceptibility to external interference. Despite these limitations, it remains a crucial component in the evaluation and treatment of cardiac conditions [15,16]. Another technique used for heart diagnosis known as Echocardiography uses ultrasound waves to create images of the heart [17]. Echocardiography is a powerful diagnostic tool with numerous benefits, including its noninvasive nature, versatility, and real-time imaging capabilities. However, it also has limitations related to operator dependence, image quality, and measurement variability [18,19,20]. Furthermore, it only gives limited time information about the heart. Cardiac MRI uses an artificially generated magnetic field [21] and radio waves to create detailed images of the heart [22]. It provides information on heart structure and function, which enables objective and functional assessment of myocardial tissue. It is not invasive, but some patients feel discomfort when performing it, specifically patients with claustrophobia [23]. Furthermore, besides being expensive, it only provide information at the time when the test is taken, i.e., it is not suitable for continuous monitoring. One of the recent techniques used to acquire information about the heart is the Cardiac CT Scan. It uses X-rays to create detailed images of the heart and its blood vessels [24,25]. It is commonly used to detect coronary artery disease and heart defects. Even though a Cardiac CT Scan is more affordable than MRI and offers noninvasive detailed imaging that can assess coronary arteries, it has many limitations, including providing limited information about the functional aspects of the heart as well as exposing patients to ionizing radiation, which has its own risks [26,27]. Furthermore, due to using contrast agents, it is harmful to those with kidney problems. In addition, some patients experience allergic reactions to these agents [26,27]. Nuclear Cardiology [28,29] involves injecting a small amount of radioactive material and using a conventional gamma camera with a sodium iodide detector and single-photon emission computed tomography (SPECT) to visualize blood flow and heart function. Another Nuclear Cardiology technique uses cardiac positron emission tomography (PET). Although Cardiac MRI and CT scans are more commonly used than Nuclear Cardiology, the latter is evolving toward an interrogation of the biology and molecular pathways [30]. While Nuclear Cardiology offers an assessment of myocardial perfusion as well as information about the effectiveness of treatment, besides being noninvasive, it comes at a high cost and has limited availability [31]. The use of radioactive material and exposing the patient to ionizing radiation can pose a risk to the patient. In [32], the authors presented a review of the modern diagnostic based on imaging techniques and their risks.

From the prior art conducted, it is clear that further noninvasive techniques remove the previously discussed limitations of the existing techniques. Different body organs generate magnetic fields. Using such fields for diagnostic purposes offers several advantages across various medical applications and provides a unique insight into the body’s internal workings [33]. Best of all, acquiring such magnetic field measurements can be performed in a noninvasive way. It is also known that the magnetic field emitted by the heart has one of the weakest signal strengths. In what follows, a prior art search will be conducted regarding the acquisition of the heart’s magnetic field.

## 2. Heart Magnetic Field Acquisition

The heart generates a weak magnetic field due to the electrical currents flowing during the heart cardiac cycle [34,35,36]. The generation of cardiac magnetic fields is rooted in the electrophysiological processes of the heart. As the heart beats, electrical currents flow through the heart muscle (myocardium) during a cardiac cycle, initiating muscular contractions that pump blood. The electrical currents flowing in the heart could be summarized by the following: First, SA Node Activation initiates the impulse followed by Atrial Depolarization, which spreads through the atria causing contraction. Next, the AV Node Delay allows for complete ventricular filling followed by the Impulse Propagation, which passes through the muscle bundle connecting the atrial and ventricular chambers of the heart, known as the “His Bundle”, the bundle branches, and the Purkinje fibers. Finally, Ventricular Depolarization causes ventricular contraction and Ventricular Repolarization leads to ventricular relaxation. This electrical activity produces both electric fields and magnetic fields around the body. The relationship between electrical currents and magnetic fields was described by Maxwell’s equations, which underpin the theoretical foundation of cardiac magnetic fields. Though much weaker than the Earth’s magnetic field [37,38], the heart’s magnetic field can be measured and used to provide diagnostics for heart diseases. The seminal work by Baule and McFee (1963) [39] on the detection of magnetic fields produced by the heart and the brain set the stage for the development of magnetocardiography (MCG) [40].

Measuring magnetic fields involves various methods, each with its own range, sensitivity, and applications. Following are some common methods that are used in the medical field.

1. **Hall Effect Sensors**
  - **Principle:** They are based on the Hall effect, which occurs when a current-carrying conductor or semiconductor is placed in a perpendicular magnetic field. This results in the generation of a voltage (the Hall voltage) perpendicular to both the current and the magnetic field.
  - **Applications:** They are used in magnetic microscopy [41] and Biomedical Applications [42,43] for detecting magnetic nanoparticles (MNPs) labeled on a biomolecule, monitoring blood pulse wave velocity, characterizing soft biological materials, controlling the syringe injection rate and eye surgery by training systems, and assisting magnetic resonance imaging (MRI) [44].
  - **Magnetic Field and Frequency Ranges:** The sensors may operate effectively in the range of $100\;\mu T/\sqrt{\mathrm{Hz}}$ to $100\;\mathrm{mT}/\sqrt{\mathrm{Hz}}$. The frequency range varies depending on the application from 1 Hz to 10 kHz.
  - **Distance from the Body:** Hall effect sensors used in medical devices are often placed close to the body, typically within a few millimeters to a few centimeters, depending on the application.
  - **Benefits:** Hall effect sensors provide a non-contact high-precision method of measurement, reducing the risk of contamination and wear. These sensors are robust and can operate in various conditions, ensuring consistent performance in medical environments. The small size of Hall effect sensors allows for them to be integrated into a wide range of medical devices, even those with space constraints.
  - **Limitations:** These sensors are sensitive to temperature fluctuations and magnetic interference and have a limited detection range, low signal-to-noise ratio, and high power consumption if required to be used in a portable or wearable device.
  - **Requires Shielding:** Yes, as it is sensitive to magnetic interference.
2. **Fluxgate Magnetometers**
  - **Principle:** They use the saturation of a ferromagnetic core in an alternating magnetic field to detect changes in the external magnetic field [45,46,47] and even down to $100\;\mathrm{fT}/\sqrt{\mathrm{Hz}}$ [38,47,48].
  - **Applications:** Fluxgate magnetometers can still be used for magnetocardiography (MCG) [49] measurements under certain conditions. In [49], the authors presented a feasibility study on the measurement of magnetocardiography (MCG) using a fluxgate magnetometer.
  - **Magnetic Field and Frequency Ranges:** The sensors may operate effectively in the range of $100\;\mathrm{mT}/\sqrt{\mathrm{Hz}}$ to $100\;\mathrm{fT}/\sqrt{\mathrm{Hz}}$. The frequency range of interest in cardiac magnetic field applications involving fluxgate magnetometers typically spans from 0.05 Hz to 100 Hz.
  - **Distance from the Body:** The distance from the body at which a fluxgate magnetometer operates can vary depending on the specific application. However, it is common for these measurements to be taken at a distance of a few centimeters to several decimeters.
  - **Benefits:** They are capable of detecting very weak magnetic fields, providing accurate measurements of both the magnitude and direction of magnetic fields, and offering a stable and reliable performance over long periods. New fluxgate magnetometers are more robust and significantly easier to handle, making them suitable for some portable applications.
  - **Limitations:** Traditional fluxgate magnetometers can be bulky, which may limit their integration into portable or compact medical devices. They generally consume more power compared to other magnetic field sensors, which can be a drawback for battery-operated medical devices [50]. These sensors can be affected by external magnetic fields and the cost of manufacturing high-precision fluxgate magnetometers can be high.
  - **Requires Shielding:** Yes, as these sensors are affected by the surrounding magnetic fields.
3. **Superconducting Quantum Interference Devices (SQUIDs)**
  - **Principle:** Introduced by Baule and McFee [36], they use superconducting loops containing Josephson junctions to detect extremely small changes in the magnetic flux. The development and refinement of the SQUIDs have significantly advanced the sensitivity and utility of MCG. SQUIDs can detect extremely faint magnetic fields, such as those generated by the heart, making them invaluable in clinical and research settings [51]. High-Tc SQUIDs are a type of Superconducting Quantum Interference Device that operate at relatively high critical temperatures (Tc), where the “high-Tc” refers to the material’s ability to become superconducting at temperatures significantly above those required by traditional superconductors [52]. While classical superconductors used in the first SQUIDs require cooling to temperatures close to absolute zero (typically below 10 Kelvin or −263.15 °C) using liquid helium, high-Tc superconductors can operate at higher temperatures, often above 77 Kelvin (−196 °C), which is the boiling point of liquid nitrogen. They still require cooling but can be made more portable than conventional low-temperature SQUIDs.
  - **Applications:** They are used in medical imaging (MRI), magnetoencephalography (MEG), magnetocardiography (MCG), magnetogastrography (MGG), and the detection of magnetic nanoparticles (MNPs).
  - **Magnetic Field and Frequency Ranges:** The sensors may operate effectively in the range of $1\;\mathrm{pT}/\sqrt{\mathrm{Hz}}$ to $100\;\mathrm{fT}/\sqrt{\mathrm{Hz}}$. The frequency range of interest in cardiac magnetic field applications involving SQUIDs typically spans from 0.5 Hz to 40 Hz.
  - **Distance from the Body:** SQUIDs are usually placed within a few centimeters of the body to minimize interference and maximize signal strength. This proximity allows for the accurate detection of magnetic fields generated by the brain or heart. SQUID sensors for MCG are positioned close to the chest, usually within a few centimeters.
  - **Benefits:** SQUIDs can detect magnetic fields as small as a few femtoteslas, making them extremely sensitive and capable of picking up subtle physiological signals. Most applications of SQUIDs, such as MEG and MCG, are noninvasive, reducing the risk and discomfort for patients, and SQUIDs provide real-time data with a high temporal resolution, which is crucial for monitoring dynamic physiological processes.
  - **Limitations:** SQUIDs require cooling to very low temperatures using liquid helium, which makes the systems complex and expensive to maintain, and operating SQUID systems requires specialized knowledge and training.
  - **Requires Shielding:** Yes, as these sensors are affected by the surrounding magnetic fields.
4. **Optically Pumped Magnetometers**
  - **Principle:** They are highly sensitive devices used to measure magnetic fields [53]. They operate on the principle of optical pumping, a process that involves the use of light (usually from a laser) to excite electrons in a gas (commonly an alkali metal, such as rubidium, cesium, or potassium) to a higher energy state. The alignment or polarization of these atoms’ spins is then perturbed by external magnetic fields. By monitoring the change in the atoms’ spin states, the magnetic field strength can be deduced with high precision.
  - **Applications:** They are used in neuroimaging and magnetoencephalography (MEG) [54,55], magnetocardiography (MCG), Muscle Activity Monitoring (Magnetomyography, MMG) [56], and medical imaging (MRI and NMR) [55].
  - **Magnetic Field and Frequency Ranges:** The sensors may operate effectively in the range of $1\;\mathrm{nT}/\sqrt{\mathrm{Hz}}$ to $1\;\mathrm{fT}/\sqrt{\mathrm{Hz}}$. The frequency range of interest in cardiac magnetic field applications involving OPMs typically spans from 0.05 Hz to 150 Hz.
  - **Distance from the Body:** OPMs can operate very close to the body, often within millimeters. This close proximity is advantageous because the magnetic field strength decreases with the square of the distance, so being closer to the heart improves the signal quality.
  - **Benefits:** Optically Pumped Magnetometers offer significant potential in advancing medical diagnostics and research by providing highly sensitive, noninvasive, and cost-effective solutions for measuring biomagnetic fields. Their applications in neuroimaging, cardiac monitoring, muscle activity analysis, and medical imaging are paving the way for new diagnostic techniques and improved patient outcomes. Unlike SQUIDs, which require cryogenic temperatures, OPMs can operate at room temperature, simplifying their use and deployment in various settings with the possibility of creating wearable systems.
  - **Limitations:** OPMs are highly sensitive to magnetic noise from the environment [57], involve complex technology, and can be costly to implement and maintain in clinical settings.
  - **Requires Shielding:** Yes, as these sensors are affected by the surrounding magnetic fields.
5. **Induction Coil Magnetometers**
  - **Principle:** They are based on Faraday’s law of electromagnetic induction, where a changing magnetic field induces a voltage in a coil.
  - **Applications:** Induction coil magnetometers are used to study biomagnetic fields generated by physiological processes in the human body. These magnetometers can measure the magnetic fields associated with brain activity. They can be employed to detect the weak magnetic fields generated by the electrical activity of the heart. This aids in studying cardiac functions and diagnosing heart conditions.
  - **Magnetic Field and Frequency Ranges:** Sensors may operate effectively in the range of $10\;\mathrm{nT}/\sqrt{\mathrm{Hz}}$ to $100\;\mathrm{pT}/\sqrt{\mathrm{Hz}}$. The frequency range of interest in cardiac magnetic field applications typically spans from 0.05 Hz to 150 Hz.
  - **Distance from the Body:** The magnetometer is placed very close to the chest, often within a few centimeters.
  - **Benefits:** These sensors provide a noninvasive means to study internal body processes. They can also detect extremely weak magnetic fields.
  - **Limitations:** Their main limitations are their sensitivity to environmental noise [58], resolution constraints, practical deployment challenges, and high costs [38]
  - **Requires Shielding:** Yes, as these sensors are affected by the surrounding magnetic fields.
6. **Magnetoresistive Sensors**
  - **Principle:** A well-known phenomenon is that the resistance of some material exhibits a change in value when in proximity of a magnetic field. A magnetoresistive sensor uses this property to measure the magnetic field of the heart [59].
  - **Applications:** Magnetoresistive sensors, particularly those utilizing the Giant Magnetoresistance (GMR) effect, have shown significant promise for medical applications due to their unique properties and advantages [60] and show the application of Giant Magnetoresistance-based micro-probes to record biological magnetic fields. GMR sensors exhibit high sensitivity, making them suitable for detecting weak magnetic fields, which is crucial in medical applications, such as magnetic resonance imaging (MRI) machines [61] and magnetoencephalography (MEG) machines [62]. In [63], the authors considered using tunnel magnetoresistance (TMR) sensors that operate at room temperature to measure weak biomagnetic fields. In [64], the authors used hybrid sensors based on Giant Magnetoresistance (GMR) to capture the magnetic signatures of the heart’s electrical activity (magnetocardiography) in healthy volunteers. The P-wave and QRS complex was clearly visible after approximately 1 min of averaging.
  - **Magnetic Field and Frequency Ranges:** The sensors may operate effectively in the range of $100\;\mu T/\sqrt{\mathrm{Hz}}$ to $100\;\mathrm{nT}/\sqrt{\mathrm{Hz}}$. Magnetoresistive sensors are capable of detecting signals across a wide frequency range, typically from near DC (0 Hz) up to several megahertz (MHz). The frequency range of interest in cardiac magnetic field applications typically spans from 0.05 Hz to 150 Hz.
  - **Distance from the Body:** The effectiveness of magnetoresistive sensors depends on their proximity to the source of the magnetic field. For medical applications, these sensors are usually placed very close to the body, often within a few centimeters (typically 1–5 cm).
  - **Benefits:** Magnetoresistive sensors, with their high sensitivity, compact size [61], versatility, compatibility with existing technologies, and noninvasive diagnostic capabilities, hold great promise for advancing medical diagnostics and improving patient care. These benefits make them a valuable tool in the fields of neurology, cardiology, and general medical diagnostics.
  - **Limitations:** Magnetoresistive sensors, while promising in various medical applications, face several significant limitations [65]. One major issue is their sensitivity to external magnetic interference, which can lead to inaccuracies in readings unless adequately shielded. Additionally, their performance can be affected by temperature fluctuations, necessitating complex temperature compensation mechanisms. Furthermore, the high cost of high-precision magnetoresistive sensors can hinder their widespread adoption.
  - **Requires Shielding:** Yes, as these sensors are affected by the surrounding magnetic fields.
7. **Atomic Magnetometers**
  - **Principle:** Similar to OPMs, atomic magnetometers measure magnetic fields by detecting the spin precession of atoms induced by magnetic fields [66]. In [38], the authors presented a detailed review on ultrasensitive magnetic field sensors for Biomedical Applications. Spin-Exchange Relaxation-Free (SERF) magnetometers represent a class of ultra-sensitive atomic magnetometers that can operate at room temperature and have the potential for miniaturization and portability. They are suitable for measuring the very weak magnetic fields [67,68] associated with the human heart [69].
  - **Applications:** They are used in magnetoencephalography (MEG) [54,55], magnetocardiography (MCG) [69], and fetal magnetocardiography (MCG) [70].
  - **Magnetic Field and Frequency Ranges:** The sensors may operate effectively in the range of $1\;\mathrm{pT}/\sqrt{\mathrm{Hz}}$ down to $1\;\mathrm{fT}/\sqrt{\mathrm{Hz}}$. Atomic magnetometers are sensitive to a broad range of frequencies, typically from DC (0 Hz) up to several kHz (kilohertz). This range makes them suitable for detecting the low-frequency magnetic fields generated by biological tissues, such as brain waves in the range of 1–100 Hz and cardiac magnetic fields.
  - **Distance from the Body:** The effectiveness of atomic magnetometers depends on their proximity to the source of the magnetic field. For medical applications, especially in neuroimaging, these devices are typically placed within a few millimeters to centimeters from the skin or scalp. For MCG, they need to be placed directly on the chest.
  - **Benefits:** Atomic magnetometers provide a combination of high sensitivity, cost-effectiveness, compactness, noninvasiveness, and precise measurement capabilities, making them highly advantageous for medical applications. These benefits position atomic magnetometers as a valuable tool in the fields of neurology and cardiology, among others, offering the potential for improved diagnostic accuracy and patient outcomes.
  - **Limitations:** Despite their potential, the use of atomic magnetometers in medical applications is constrained by their sensitivity to environmental noise, their operational complexity, and the need for precise temperature control. Sometimes performance is limited due to atomic vapor [66].
  - **Requires Shielding:** To achieve high sensitivity, atomic magnetometers often require operation in magnetically shielded environments to minimize interference from external magnetic sources.
8. **Search Coil Magnetometers**
  - **Principle:** They measure the time derivative of the magnetic field using a coil of wire [38].
  - **Applications:** Search coil magnetometers are highly sensitive and can detect very weak magnetic fields, which is crucial for applications like magnetoencephalography (MEG) and magnetocardiography (MCG).
  - **Magnetic Field and Frequency Ranges:** The sensors may operate effectively in the range of $1\;\mathrm{nT}/\sqrt{\mathrm{Hz}}$ down to $100\;\mathrm{fT}/\sqrt{\mathrm{Hz}}$. The operational frequency range for search coil magnetometers in medical applications typically spans from a few millihertz to several kilohertz. This range is particularly suited for capturing the low-frequency biomagnetic signals generated by physiological processes, like the cardiac cycle.
  - **Distance from the Body:** The distance from the body at which these magnetometers operate effectively can vary. For applications like magnetocardiography, they are typically placed close to the body, often within a few centimeters, to capture the weak magnetic fields generated by the heart. The exact distance depends on the required spatial resolution and the magnetic field strength.
  - **Benefits:** These magnetometers provide a noninvasive method to monitor and diagnose various medical conditions due to their high sensitivity and capability to detect weak magnetic fields. Compared to other high-sensitivity magnetic field sensors like SQUIDs, search coil magnetometers can be more cost-effective as they do not require cryogenic cooling and are simpler to operate and maintain.
  - **Limitations:** Their limitations include susceptibility to environmental noise [71], temperature sensitivity, complex calibration needs, size, and frequency range restrictions.
  - **Requires Shielding:** Yes, as these sensors are affected by the surrounding magnetic fields.
9. **Proton Precession Magnetometers**
  - **Principle:** The operating principle of a proton magnetometer is based on nuclear magnetic resonance (NMR). In a PPM, the strength of the magnetic field is determined by measuring the precession frequency of hydrogen protons within the device, which aligns with the external magnetic field [72].
  - **Applications:** PPMs can be integrated into MRI systems to improve the measurement of magnetic fields and enhance imaging quality [73].
  - **Magnetic Field and Frequency Ranges:** The sensors may operate effectively in the range of $1\;\mathrm{mT}/\sqrt{\mathrm{Hz}}$ down to $100\;\mathrm{nT}/\sqrt{\mathrm{Hz}}$. In medical PPM applications where magnetic fields are much weaker, the corresponding frequencies would be in the kilohertz (kHz) range.
  - **Distance from the Body:** PPMs need to be sensitive and placed close to the chest.
  - **Benefits:** Proton Precession Magnetometers hold significant potential in medical applications due to their high sensitivity, noninvasive nature, and portability, making them easy to transport and deploy in various environments.
  - **Limitations:** Their sensitivity to external factors, lower sampling rate, and complex calibration requirements are notable limitations that need to be addressed [72].
  - **Requires Shielding:** Yes, as these sensors are affected by the surrounding magnetic fields.
10. **Magnetoelectric Sensors**
  - **Principle:** These sensors depend on magnetoelectric (ME) composites that show the ME response at room temperature. When a magnetic field is applied to ME composites, an electric charge across the piezoelectric phase is induced due to the piezoelectricity that results from the deformation produced by the ferromagnetic component due to the magnetostriction that is transferred to the ferroelectric component via interfacial bonding [74].
  - **Applications:** A pilot study on using these sensors for MCG is presented in [75]. In [76], the authors discussed using ME sensor systems for cardiological applications. They combined the obtained readings with an ECG and averaging techniques to improve the signal-to-noise ratio (SNR).
  - **Magnetic Field and Frequency Ranges:** It is $20\;\mathrm{pT}/\sqrt{\mathrm{Hz}}$ [77]. It can reach $1\;\mathrm{fT}/\sqrt{\mathrm{Hz}}$ [78] at mechanical resonance. The frequency range for ME sensors in medical applications spans from low frequencies (e.g., 0.1 Hz in neuronal activity) to high frequencies (e.g., MHz in implantable devices).
  - **Distance from the Body:** The operational distance varies from a few millimeters (for implantable devices) to several centimeters (for noninvasive measurements, like MEG or MCG).
  - **Benefits:** These sensors consume little power, are highly sensitivities, and have large linear dynamic ranges.
  - **Limitations:** The performance of ME sensors can be affected by temperature changes, which may require compensation or stabilization techniques.
  - **Requires Shielding:** To obtain highly sensitive data readings, the sensors have to be in a shielded area. For example, in [75], the experiments were conducted in a shielded room.

Each of these methods/techniques offers unique advantages and is selected based on the required sensitivity, range, and application context. The size of the sensor is an important aspect when deciding whether this technology could be used or not if portability is important. For example, the portable measurement of magnetocardiography (MCG) holds significant importance such as broadening diagnostic reach outside of specialized cardiac centers, making cardiac monitoring accessible in remote or underserved areas. Patients with chronic heart conditions can also be monitored at home, improving patient comfort and reducing hospital visits. Real-time data can help in adjusting treatments promptly, preventing the progression of heart conditions that would otherwise require more intensive and expensive interventions. The development of portable devices for MCG measurement is an active area of research, focusing on improving sensitivity, reducing interference from external magnetic fields, and making the devices easier to use in a clinical setting or for remote monitoring. The availability of such devices could revolutionize cardiac care, allowing for more widespread and convenient monitoring of heart health. The development of wearable MCG systems could further enhance patient monitoring, integrating seamlessly with other health monitoring.

Table 1 shows the different methods used for measuring the magnetic field and their range of measurement as well as their applications in the medical field.

**Figure 2:**
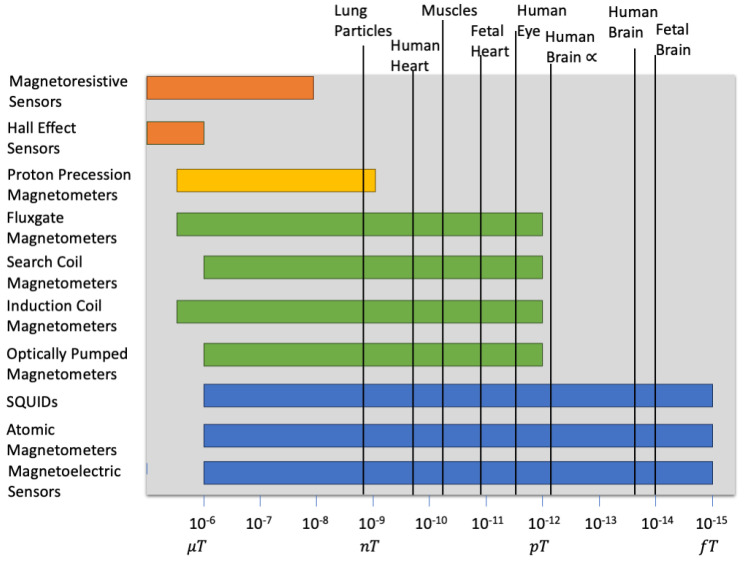
The magnetic fields of different body organs and the devices capable of sensing them [69].

Figure 2 shows the biomagnetic fields generated from different body organs, and it also shows the range of the different magnetic sensors used in medical applications. It is clear that there are many devices capable of measuring the magnetic field of the heart. The main problem is the noise due to the many environmental fields around the body, specifically the magnetic fields due to electronic devices. This paper focuses on the measurement of the magnetic field generated by the heart and its capability to be used to diagnose different heart diseases.

## 3. Heart Disease Diagnosis Using MCG

Magnetocardiography (MCG) has seen significant advancements and applications in medical diagnostics. This technology offers a radiation-free alternative to traditional cardiac diagnostic tools, with unique benefits in certain clinical scenarios. Below are some of the current applications of MCG in medical diagnostics:

- **Ischemic Heart Disease Detection:** MCG has been found to be particularly useful in the early detection of ischemic heart disease. It can identify ischemic changes in the heart that might not be apparent in a conventional electrocardiogram (ECG), especially in cases of microvascular dysfunction or in patients with non-obstructive coronary artery disease [79]. The basis for using MCG in the detection of ischemic heart disease lies in its ability to detect subtle changes in the cardiac magnetic field patterns associated with ischemia. Ischemia, a condition characterized by reduced blood flow to the heart muscle, can lead to alterations in the myocardial electrical properties, which in turn affects the heart’s magnetic field. MCG is sensitive to these changes, even in the early stages of ischemia. There are many advantages for using MCG over conventional methods. MCG has been shown to have a higher sensitivity in detecting ischemic changes, particularly in patients with non-obstructive coronary artery disease or microvascular dysfunction [80]. Unlike angiography or other invasive diagnostic methods, MCG is completely noninvasive and does not involve exposure to radiation, making it safer for repeated use. Several studies have highlighted the effectiveness of MCG in detecting ischemic heart disease [81]. The future of ischemic heart disease detection using MCG looks promising, with ongoing research focused on enhancing the technology’s sensitivity and specificity. Studies indicate that MCG can differentiate between patients with CAD (coronary artery disease) and healthy controls, offering diagnostic accuracy superior to traditional tests like an EKG in certain scenarios. MCG’s ability to detect myocardial ischemia in patients with a normal EKG and biomarker has been highlighted in [80].
  Efforts are also underway to make MCG more accessible and cost-effective for widespread clinical use. Furthermore, combining MCG data with machine learning algorithms could improve diagnostic accuracy and personalize patient care as it has done with EEG, CT, PET, and MRI [82,83,84].
- **Cardiac Arrhythmias:** Cardiac arrhythmias, which are irregular heartbeats caused by abnormal electrical conduction or the firing of electrical impulses in the heart, produce distinct magnetic field patterns. MCG can detect these patterns, enabling the diagnosis and analysis of arrhythmias with a high degree of sensitivity. The technology’s ability to noninvasively map the heart’s magnetic field in detail allows for the identification of arrhythmic sources and pathways [36] that may not be apparent, with ECG contributing to more accurate diagnosis and treatment planning for conditions, such as atrial fibrillation [85], ventricular tachycardia, and others [86]. Research into MCG’s application in arrhythmia diagnosis is ongoing, with studies focusing on improving the technology’s accuracy, portability, and cost-effectiveness.
- **Risk Stratification:** MCG technology has shown potential in risk stratification among patients with heart failure, identifying those at higher risk of arrhythmias or sudden cardiac death. By analyzing the heart’s magnetic signals, MCG can detect subtle abnormalities in cardiac function that are indicators of a poor prognosis, such as heterogeneities in repolarization, which are not easily detectable with conventional electrocardiography (ECG) [87,88].
- **Fetal Cardiac Monitoring:** One of the most promising applications of MCG is in fetal cardiac monitoring [89]. MCG can be used to assess the fetal heart’s electrophysiological properties, detecting congenital heart defects and arrhythmias in early pregnancy without the risks associated with invasive procedures [90,91].
- **Pharmacological Studies:** MCG has been employed in pharmacological studies to evaluate the effects of drugs on cardiac electrophysiology, particularly for monitoring the QT interval, the time taken for ventricular depolarization and repolarization. This application is crucial for both clinical trials and post-market surveillance of medications [92].

Magnetocardiography offers a valuable addition to the toolkit of noninvasive cardiac diagnostics, with applications ranging from the detection of ischemic heart disease and arrhythmias to fetal monitoring and drug safety assessments. Its unique ability to provide detailed information about the heart’s electrical activity, without the need for exposure to radiation, positions MCG as a promising technology for advancing cardiac care. Future developments and wider availability of MCG systems are expected to further expand its use in clinical practice.

Because ECG is the most common technique used to diagnose common heart diseases, a comparison between it and MCG would highlight the main difference between the two techniques, as in Table 2.

From Table 2, one can say that ECG is widely used for diagnosing arrhythmias, myocardial infarction, and other cardiac conditions. It is known for being widely available and cost-effective. However, ECG has limitations in detecting subtle ischemic changes, particularly in the early stages of heart disease or in cases involving microvascular dysfunction. The electrical signals measured by ECG can sometimes be insufficient to detect all types of heart abnormalities, leading to potential false negatives in some scenarios [93].

On the other hand, MCG is particularly useful for detecting ischemic heart disease, including instances with microvascular dysfunction and non-obstructive coronary artery disease. MCG offers higher sensitivity to subtle ischemic changes compared to ECG, making it capable of identifying abnormalities that might not be detectable using ECG. Additionally, MCG is noninvasive and does not involve radiation exposure, which makes it safe for repeated uses.

In summary, while ECG is a cost-effective and widely accessible tool for diagnosing a range of heart conditions, it has limitations in sensitivity and specificity, especially for early stage ischemia. MCG, with its higher sensitivity to subtle magnetic changes in the heart, offers a valuable complementary approach for detecting ischemic heart disease and other cardiac abnormalities. However, the higher cost, complexity, and sensitivity to environmental noise of MCG technology poses significant challenges to its widespread adoption. As advancements continue, MCG has the potential to become a more prevalent tool in cardiac diagnostics, particularly for cases where ECG falls short.

This comparison highlights the fact that while ECG is the most commonly used for routine cardiac monitoring, MCG offers higher sensitivity and spatial resolution, which can be particularly useful in specialized applications where these factors are critical. However, MCG’s higher cost and equipment requirements limit its widespread use compared to ECG.

## 4. Recommendations and Future Work

Table 3 summarizes the common issues faced by different magnetic sensor technologies in measuring the heart’s magnetic field, highlighting challenges such as sensitivity, environmental interference, and operational complexity. Almost all of the sensors currently used for MCG require shielding to avoid any noise from the surroundings, which complicates the deployment of these sensors in medical facilities. Developing more advanced magnetic sensor technology for heart magnetic field measurement is essential for improving diagnostic accuracy, enhancing patient comfort, and enabling early detection and intervention in cardiac care. These advancements could lead to better management of heart diseases and overall better healthcare outcomes. The need also for portable and wearable devices is increasing as the cost for hospitalization is getting higher. The problems listed in Table 3 extend to portable and wearable devices, as most of the current sensing technologies are bulky, sensitive to noise and temperature, and require complex calibration and setup. All of these problems are affecting the use of MCG as a diagnostic tool for heart diseases even though it is considered one of the most informative tools if used.

## 5. Conclusions

In conclusion, harnessing the heart’s magnetic field for advanced diagnostic techniques represents a significant leap forward in cardiac care. Magnetocardiography (MCG) offers unparalleled sensitivity and specificity in detecting ischemic changes and other subtle cardiac abnormalities, outperforming traditional electrocardiography (ECG) in many aspects. Despite challenges such as environmental noise sensitivity and higher costs, ongoing research in magnetic sensor technology and noise reduction strategies [94,95] holds promise for making MCG more accessible and practical. As research progresses, MCG is poised to become an indispensable tool in early and accurate cardiac diagnostics, ultimately enhancing patient outcomes and revolutionizing heart disease management, especially when it comes to continuous monitoring using portable sensor-based heart magnetic fields.

Future developments may include integrating MCG findings with other diagnostic modalities, like imaging techniques, to enhance the overall understanding and treatment of arrhythmias. Moreover, advancements in signal processing and machine learning could further refine the diagnostic capabilities of MCG, allowing for automated identification and classification of complex arrhythmias.

## Figures and Tables

**Table 1:** Comparison of magnetic sensor technologies in medical applications.

Magnetic Sensor Technology	Measurement Range	Medical Applications
Hall Effect Sensors	$100\;\mathrm{mT}/\sqrt{\mathrm{Hz}}$ to $100\;\mu T/\sqrt{\mathrm{Hz}}$	Magnetic microscopy [41], detecting magnetic nanoparticles (MNPs)
Fluxgate Magnetometers	$100\;\mathrm{fT}/\sqrt{\mathrm{Hz}}$ to $100\;\mathrm{mT}/\sqrt{\mathrm{Hz}}$	Biomagnetic field detection, magnetoencephalography (MEG)
Superconducting Quantum Interference Devices (SQUIDs)	$100\;\mathrm{fT}/\sqrt{\mathrm{Hz}}$ to $1\;\mathrm{pT}/\sqrt{\mathrm{Hz}}$	Magnetoencephalography (MEG), magnetocardiography (MCG), MRI
Optically Pumped Magnetometers	$1\;\mathrm{fT}/\sqrt{\mathrm{Hz}}$ to $1\;\mathrm{nT}/\sqrt{\mathrm{Hz}}$	Magnetoencephalography (MEG), magnetocardiography (MCG)
Induction Coil Magnetometers	$100\;\mathrm{pT}/\sqrt{\mathrm{Hz}}$ to $10\;\mathrm{nT}/\sqrt{\mathrm{Hz}}$	Detection of neural and cardiac magnetic fields
Magnetoresistive Sensors	$100\;\mathrm{nT}/\sqrt{\mathrm{Hz}}$ to $100\;\mu T/\sqrt{\mathrm{Hz}}$	Magnetic resonance imaging (MRI), biosensors
Atomic Magnetometers	$1\;\mathrm{fT}/\sqrt{\mathrm{Hz}}$ to $1\;\mathrm{pT}/\sqrt{\mathrm{Hz}}$	Magnetoencephalography (MEG), magnetocardiography (MCG)
Search Coil Magnetometers	$100\;\mathrm{fT}/\sqrt{\mathrm{Hz}}$ to $1\;\mathrm{nT}/\sqrt{\mathrm{Hz}}$	Magnetic field mapping in medical research
Proton Precession Magnetometers	$100\;\mathrm{nT}/\sqrt{\mathrm{Hz}}$ to $1\;\mathrm{mT}/\sqrt{\mathrm{Hz}}$	Magnetocardiography (MCG), fetal monitoring, MRI
Magnetoelectric Sensors	$1\;\mathrm{fT}/\sqrt{\mathrm{Hz}}$ [77,78] to $20\;\mathrm{pT}/\sqrt{\mathrm{Hz}}$ [77]	Magnetocardiography (MCG)

**Table 2:** Comparison between MCG and ECG.

Feature	Magnetocardiography (MCG)	Electrocardiography (ECG)
Principle of operation	Measures the magnetic fields generated by the heart’s electrical activity [92].	Measures the electrical activity of the heart via skin electrodes.
Sensitivity	High sensitivity to small, intracardiac electrical events [80,85].	Lower sensitivity to small, intracardiac signals compared to MCG.
Spatial resolution	High; can provide detailed mapping of cardiac electrical activity.	Relatively lower spatial resolution.
Invasiveness	Noninvasive and does not require any type of contact to the patient’s body.	Noninvasive, but some patients find it uncomfortable as it touches their skin.
Equipment	Requires specialized equipment, such as SQUID sensors, often housed in magnetically shielded rooms [92].	Standardized and widely available equipment.
Cost	Generally more expensive due to specialized equipment and facility requirements.	Less expensive and more widely accessible.
Use cases	Particularly useful in detecting and analyzing arrhythmias, ischemic heart disease, and fetal heart conditions.	Broadly used for routine cardiac assessments and diagnosing arrhythmias, myocardial infarction, and other heart conditions.
Signal interference	Less susceptible to muscle and movement artifacts due to the nature of magnetic field measurement.	More susceptible to noise from muscle movements and electrode displacement.
Availability	Less widely available; mainly used in research settings and specialized clinical centers.	Widely available in hospitals, clinics, and even as portable devices for home use.
Patient comfort	Generally comfortable, but requires lying still in a magnetically shielded room.	Generally comfortable, but adhesive electrodes may cause minor skin irritation for some patients.

**Table 3:** Problems facing different magnetic sensor technologies in medical applications.

Magnetic Sensor Technology	Problems
Hall Effect Sensors	Sensitive to temperature fluctuations, magnetic interference (requires shielding), has a limited detection range, low signal-to-noise ratio, high power consumption.
Fluxgate Magnetometers	Susceptible to temperature fluctuations, requires complex calibration, bulkiness can limit portability.
Superconducting Quantum Interference Devices (SQUIDs)	Requires cryogenic cooling, high operational costs, complex setup and maintenance.
Optically Pumped Magnetometers	Sensitivity to light and temperature fluctuations, requires optical components, potentially bulky.
Induction Coil Magnetometers	Sensitivity to external noise, lower spatial resolution, bulky and less portable.
Atomic Magnetometers	Requires magnetic shielding, sensitivity to environmental noise, complex operation and maintenance.
Search Coil Magnetometers	Susceptibility to electromagnetic noise, requires specialized environments or shielding, temperature sensitivity.
Proton Precision Magnetometers	Sensitivity to external factors, lower sampling rate, complex calibration requirements.
Magnetoelectric Sensors	Sensitivity to temperature changes.
Magnetoresistive Sensors	Sensitivity to external magnetic interference, sensitivity to temperature fluctuations, high cost.

## Data Availability

No new data were created or analyzed in this study. Data sharing is not applicable to this article.

## References

[1] Federation W.H. World Heart Report 2023. https://world-heart-federation.org/wp-content/uploads/World-Heart-Report-2023.pdf.

[2] World Health Organization Cardiovascular Diseases. https://www.who.int/news-room/fact-sheets/detail/cardiovascular-diseases-(cvds).

[3] Kim H.C. (2021). Epidemiology of cardiovascular disease and its risk factors in Korea. Glob. Health Med..

[4] England P.H. Health Matters: Preventing Cardiovascular Disease. https://www.gov.uk/government/publications/health-matters-preventing-cardiovascular-disease/health-matters-preventing-cardiovascular-disease.

[5] World Health Organization Cardiovascular Diseases. https://www.who.int/health-topics/cardiovascular-diseases#tab=tab_1.

[6] Rafie N., Kashou A.H., Noseworthy P.A. (2021). ECG Interpretation: Clinical Relevance, Challenges, and Advances. Hearts.

[7] Asch F.M., Shah S., Rattin C., Swaminathan S., Fuisz A., Lindsay J. (2006). Lack of sensitivity of the electrocardiogram for detection of old myocardial infarction: A cardiac magnetic resonance imaging study. Am. Heart J..

[8] Roguin A. (2006). Rene Theophile Hyacinthe Laënnec (1781–1826): The man behind the stethoscope. Clin. Med. Res..

[9] Singh S., Goyal A. (2007). The origin of echocardiography: A tribute to Inge Edler. Tex. Heart Inst. J..

[10] (2021). Utility and Limitations of the Surface ECG. Clinical Electrocardiography.

[11] Barold S.S. (2003). Willem Einthoven and the birth of clinical electrocardiography a hundred years ago. Card. Electrophysiol. Rev..

[12] West J.J., Simpson A.G. (2009). What are the Limitations of the ECG in Clinical Practice?. Critical Decisions in Emergency and Acute Care Electrocardiography.

[13] Patel V., Danish M., Monteleone C. (2018). All Roads “Lead” to Anaphylaxis: Hypersensitivity to Electrocardiogram Leads. Ann. Allergy Asthma Immunol..

[14] Mubarik I.A. Holter Monitor. https://www.ncbi.nlm.nih.gov/books/NBK538203/.

[15] Galli A., Ambrosini F., Lombardi F. (2016). Holter Monitoring and Loop Recorders: From Research to Clinical Practice. Arrhythmia Electrophysiol. Rev..

[16] Krish Tangella A.E., Warren A. Holter Monitor: Exploring the Applications, Procedure, and Analysis of Continuous ECG Monitoring. https://www.dovemed.com/health-topics/focused-health-topics/holter-monitor-exploring-applications-procedure-and-analysis-continuous-ecg-monitoring.

[17] Pohost G.M. (2008). The History of Cardiovascular Magnetic Resonance. JACC Cardiovasc. Imaging.

[18] Potter A., Pearce K., Hilmy N. (2019). The benefits of echocardiography in primary care. Br. J. Gen. Pract..

[19] Lancellotti P., Price S., Edvardsen T., Cosyns B., Neskovic A.N., Dulgheru R., Flachskampf F.A., Hassager C., Pasquet A., Gargani L. (2014). The use of echocardiography in acute cardiovascular care: Recommendations of the European Association of Cardiovascular Imaging and the Acute Cardiovascular Care Association. Eur. Heart J. Cardiovasc. Imaging.

[20] Marwick T.H. (2015). The Role of Echocardiography in Heart Failure. J. Nucl. Med..

[21] Rajiah P.S., François C.J., Leiner T. (2023). Cardiac MRI: State of the Art. Radiology.

[22] Seetharam K., Lerakis S. (2019). Cardiac magnetic resonance imaging: The future is bright. F1000Research.

[23] Siddiqui T.A., Chamarti K.S., Tou L.C., Demirjian G.A., Noorani S., Zink S., Umair M. (2022). The Merits, Limitations, and Future Directions of Cost-Effectiveness Analysis in Cardiac MRI with a Focus on Coronary Artery Disease: A Literature Review. J. Cardiovasc. Dev. Dis..

[24] Nikolaou K., Alkadhi H., Bamberg F., Leschka S. (2011). MRI and CT in the diagnosis of coronary artery disease: Indications and applications. Insights Imaging.

[25] Ritman E.L. (2003). Cardiac computed tomography imaging: A history and some future possibilities. Cardiol. Clin..

[26] Clinic C. Cardiac Computed Tomography (CT) Scan. https://my.clevelandclinic.org/health/diagnostics/16834-cardiac-computed-tomography.

[27] Prat-Gonzalez S., Sanz J., Garcia M.J. (2008). Cardiac CT: Indications and Limitations. J. Nucl. Med. Technol..

[28] Anagnostopoulos C., Underwood R. (2012). Nuclear cardiology. Clin. Med..

[29] Malek H., Maleki M., Alizadehasl A., Haghjoo M. (2022). Chapter 9—Nuclear Cardiology. Practical Cardiology.

[30] Werner R.A., Thackeray J.T., Diekmann J., Weiberg D., Bauersachs J., Bengel F.M. (2020). The Changing Face of Nuclear Cardiology: Guiding Cardiovascular Care Toward Molecular Medicine. J. Nucl. Med. Off. Publ. Soc. Nucl. Med..

[31] Underwood S.R., de Bondt P., Flotats A., Marcasa C., Pinto F., Schaefer W., Verberne H.J. (2014). The current and future status of nuclear cardiology: A consensus report. Eur. Heart J. Cardiovasc. Imaging.

[32] Hussain S., Mubeen I., Ullah N., Shah S.S.U.D., Khan B.A., Zahoor M., Ullah R., Khan F.A., Sultan M.A. (2022). Modern Diagnostic Imaging Technique Applications and Risk Factors in the Medical Field: A Review. Biomed Res. Int..

[33] Rotundo S., Brizi D., Flori A., Giovannetti G., Menichetti L., Monorchio A. (2022). Shaping and Focusing Magnetic Field in the Human Body: State-of-the Art and Promising Technologies. Sensors.

[34] Tenforde T.S. (2005). Magnetically induced electric fields and currents in the circulatory system. Prog. Biophys. Mol. Biol..

[35] Verywellhealth The Heart’s Electrical System: Anatomy and Function. https://www.verywellhealth.com/cardiac-electrical-system-how-the-heart-beats-1746299.

[36] Baule G.M., McFee R. (1970). The magnetic heart vector. Am. Heart J..

[37] Turutin A.V., Kubasov I.V., Kislyuk A.M., Kuts V.V., Malinkovich M.D., Parkhomenko Y.N., Sobolev N.A. (2022). Ultra-Sensitive Magnetoelectric Sensors of Magnetic Fields for Biomedical Applications. Nanobiotechnol. Rep..

[38] Murzin D., Mapps D.J., Levada K., Belyaev V., Omelyanchik A., Panina L., Rodionova V. (2020). Ultrasensitive Magnetic Field Sensors for Biomedical Applications. Sensors.

[39] Baule G. (1963). Detection of the magnetic field of the heart. Am. Heart J..

[40] Plonsey R. (2007). Bioelectricity: A Quantitative Approach.

[41] Sinimbu L.I., Gutierrez F.V., Lima C.D., Sommer R.L., Silva B.G., Serna J.D.P., Luz-Lima C., Bruno A.C., Araújo J.F. (2024). Magnetic microscopy using Hall effect sensors biased with pulsed currents. J. Magn. Magn. Mater..

[42] Uddin S.M., Sayad A., Chan J., Skafidas E., Kwan P. (2022). Design and Optimisation of Elliptical-Shaped Planar Hall Sensor for Biomedical Applications. Biosensors.

[43] Lapicki A., Sanbonsugi H., Yamamura T., Matsushita N., Abe M., Narimatsu H., Handa H., Sandhu A. (2005). Functionalization of micro-Hall effect sensors for biomedical applications utilizing superparamagnetic beads. IEEE Trans. Magn..

[44] Fan H., Wang J., Feng Q., Hu Q., Zuo S., Nabaei V., Heidari H. (2021). Detection techniques of biological and chemical Hall sensors. RSC Adv..

[45] Jeng J.T., Lu C.C., Ku H.W., Huang B.R., Chia M.H., Trinh X.T. (2019). Three-Axis Micofluxgate With a Fluxguide. IEEE Trans. Magn..

[46] Miles D.M., Ciurzynski M., Barona D., Narod B.B., Bennest J.R., Kale A., Lessard M., Milling D.K., Larson J., Mann I.R. (2019). Low-noise permalloy ring cores for fluxgate magnetometers. Geosci. Instrum. Methods Data Syst..

[47] Elrefai A.L., Sasada I., Harada S. (2015). Gradiometer and Magnetometer Integration Using a Pair of Fundamental Mode Orthogonal Fluxgate Sensor Heads. IEEE Trans. Magn..

[48] Janosek M., Grosz A., Haji-Sheikh M.J., Mukhopadhyay S.C. (2017). Parallel Fluxgate Magnetometers. High Sensitivity Magnetometers.

[49] Sengottuvel S., Sharma A., Biswal D., Khan P.F., Swain P.P., Patel R., Gireesan K. (2018). Feasibility study on measurement of magnetocardiography (MCG) using fluxgate magnetometer. AIP Conf. Proc..

[50] Lu C.C., Huang J., Chiu P.K., Chiu S.L., Jeng J.T. (2014). High-Sensitivity Low-Noise Miniature Fluxgate Magnetometers Using a Flip Chip Conceptual Design. Sensors.

[51] Cohen D. (1968). Magnetoencephalography: Evidence of magnetic fields produced by alpha-rhythm currents. Science.

[52] Erné S.N., Lehmann J., Weinstock H. (1996). Magnetocardiography, an introduction. SQUID Sensors: Fundamentals, Fabrication and Applications.

[53] Budker D. (2007). Optical magnetometry. Nat. Phys..

[54] Wittevrongel B., Holmes N., Boto E., Hill R., Rea M., Libert A., Khachatryan E., Van Hulle M.M., Bowtell R., Brookes M.J. (2021). Practical real-time MEG-based neural interfacing with optically pumped magnetometers. BMC Biol..

[55] Zahran S., Mahmoudzadeh M., Wallois F., Betrouni N., Derambure P., Le Prado M., Palacios-Laloy A., Labyt E. (2022). Performance Analysis of Optically Pumped 4He Magnetometers vs. Conventional SQUIDs: From Adult to Infant Head Models. Sensors.

[56] Sometti D., Semeia L., Baek S., Chen H., Righetti G., Dax J., Kronlage C., Kirchgässner M., Romano A., Heilos J. (2021). Muscle Fatigue Revisited—Insights From Optically Pumped Magnetometers. Front. Physiol..

[57] Xiang J., Yu X., Bonnette S., Anand M., Riehm C.D., Schlink B., Diekfuss J.A., Myer G.D., Jiang Y. (2023). Improved Biomagnetic Signal-To-Noise Ratio and Source Localization Using Optically Pumped Magnetometers with Synthetic Gradiometers. Brain Sci..

[58] Marmugi L., Renzoni F. (2020). Electromagnetic Induction Imaging with Atomic Magnetometers: Progress and Perspectives. Appl. Sci..

[59] Sensors R. Magnetoresistive Sensors. https://www.rechner-sensors.com/en/documentations/knowledge/magnetoresistive-sensors.

[60] Barbieri F., Trauchessec V., Caruso L., Trejo-Rosillo J., Telenczuk B., Paul E., Bal T., Destexhe A., Fermon C., Pannetier-Lecoeur M. (2016). Local recording of biological magnetic fields using Giant Magneto Resistance-based micro-probes. Sci. Rep..

[61] Electricity Magnetism.org Magnetoresistive Sensors. https://www.electricity-magnetism.org/magnetoresistive-sensors/.

[62] Kanno A., Nakasato N., Oogane M., Fujiwara K., Nakano T., Arimoto T., Matsuzaki H., Ando Y. (2022). Scalp attached tangential magnetoencephalography using tunnel magneto-resistive sensors. Sci. Rep..

[63] Fujiwara K., Oogane M., Kanno A., Imada M., Jono J., Terauchi T., Okuno T., Aritomi Y., Morikawa M., Tsuchida M. (2018). Magnetocardiography and magnetoencephalography measurements at room temperature using tunnel magneto-resistance sensors. Appl. Phys. Express.

[64] Pannetier-Lecoeur M., Parkkonen L., Sergeeva-Chollet N., Polovy H., Fermon C., Fowley C. (2011). Magnetocardiography with sensors based on giant magnetoresistance. Appl. Phys. Lett..

[65] Cubells-Beltran M., Reig C., Martos J., Torres J., Soret J. (2011). Limitations of Magnetoresistive Current Sensors in Industrial Electronics Applications. Int. Rev. Electr. Eng..

[66] Bai X., Wen K., Peng D., Liu S., Luo L. (2023). Atomic magnetometers and their application in industry. Front. Phys..

[67] Ma Y., Qiao Z., Yu M., Wang Y., Chen Y., Luo G., Yang P., Lin Q., Zhao L., Zhang Y. (2022). Single-beam integrated hybrid optical pumping spin exchange relaxation free magnetometer for biomedical applications. Appl. Phys. Lett..

[68] Savukov I.M., Grosz A., Haji-Sheikh M.J., Mukhopadhyay S.C. (2017). Spin Exchange Relaxation Free (SERF) Magnetometers. High Sensitivity Magnetometers.

[69] Wyllie R., Kauer M., Smetana G.S., Wakai R.T., Walker T.G. (2012). Magnetocardiography with a modular spin-exchange relaxation-free atomic magnetometer array. Phys. Med. Biol..

[70] Wakai R.T. (2014). The atomic magnetometer: A new era in biomagnetism. AIP Conf. Proc..

[71] Nourmohammadi Abadchi A., Feiz S., Asteraki M. (2014). Investigation of Noise Reduction and SNR Enhancement in Search Coil Magnetometers at Low Frequencies. arXiv.

[72] Electricity Magnetism.org Proton Precession Magnetometer. https://www.electricity-magnetism.org/proton-precession-magnetometer/.

[73] Ayon A.I. (2018). Proton Precession Magnetometer. Bachelor’s Thesis.

[74] Wang Y., Li J., Viehland D. (2014). Magnetoelectrics for magnetic sensor applications: Status, challenges and perspectives. Mater. Today.

[75] Elzenheimer E., Hayes P., Thormählen L., Engelhardt E., Zaman A., Quandt E., Frey N., Höft M., Schmidt G. (2023). Investigation of Converse Magnetoelectric Thin-Film Sensors for Magnetocardiography. IEEE Sens. J..

[76] Reermann J., Durdaut P., Salzer S., Demming T., Piorra A., Quandt E., Frey N., Höft M., Schmidt G. (2018). Evaluation of magnetoelectric sensor systems for cardiological applications. Measurement.

[77] Bichurin M., Petrov R., Sokolov O., Leontiev V., Kuts V., Kiselev D., Wang Y. (2021). Magnetoelectric Magnetic Field Sensors: A Review. Sensors.

[78] Viehland D., Wuttig M., McCord J., Quandt E. (2018). Magnetoelectric magnetic field sensors. MRS Bull..

[79] Yamada S., Yamaguchi I. (2005). agnetocardiograms in clinical medicine: Unique information on cardiac ischemia, arrhythmias, and fetal diagnosis. Intern. Med..

[80] Her A.Y., Dischl D., Kim Y.H., Kim S.W., Shin E.S. (2023). Magnetocardiography for the detection of myocardial ischemia. Front. Cardiovasc. Med..

[81] Brisinda D., Caristo M.E., Fenici R. (2007). Longitudinal study of cardiac electrical activity in anesthetized guinea pigs by contactless magnetocardiography. Physiol. Meas..

[82] Krishnan G., Singh S., Pathania M., Gosavi S., Abhishek S., Parchani A., Dhar M. (2023). Artificial intelligence in clinical medicine: Catalyzing a sustainable global healthcare paradigm. Front. Artif. Intell..

[83] Pinto-Coelho L. (2023). How Artificial Intelligence Is Shaping Medical Imaging Technology: A Survey of Innovations and Applications. Bioengineering.

[84] Khalifa M., Albadawy M. (2024). AI in diagnostic imaging: Revolutionising accuracy and efficiency. Comput. Methods Programs Biomed. Update.

[85] Yoshida K., Ogata K., Inaba T., Nakazawa Y., Ito Y., Yamaguchi I., Kandori A., Aonuma K. (2015). Ability of magnetocardiography to detect regional dominant frequencies of atrial fibrillation. J. Arrhythmia.

[86] Brisinda D., Fenici P., Fenici R. (2023). Clinical magnetocardiography: The unshielded bet—Past, present, and future. Front. Cardiovasc. Med..

[87] Grimm W., Glaveris C., Hoffmann J., Menz V., Müller H.H., Hufnagel G., Maisch B. (2000). RArrhythmia risk stratification in idiopathic dilated cardiomyopathy based on echocardiography and 12-lead, signal-averaged, and 24-hour holter electrocardiography. Am. Heart J..

[88] Korhonen P., Väänänen H., Mäkijärvi M., Katila T., Toivonen L. (2001). Repolarization abnormalities detected by magnetocardiography in patients with dilated cardiomyopathy and ventricular arrhythmias. J. Cardiovasc. Electrophysiol..

[89] Strand S.A., Strasburger J.F., Wakai R.T. (2019). Fetal magnetocardiogram waveform characteristics. Physiol. Meas..

[90] Strasburger J. (2008). Agnetocardiography for fetal arrhythmias. Heart Rhythm..

[91] Comani S., Liberati M., Mantini D., Gabriele E., Brisinda D., Di Luzio S., Fenici R., Luca Romani G. (2004). Characterization of Fetal Arrhythmias by Means of Fetal Magnetocardiography in Three Cases of Difficult Ultrasonographic Imaging. Pacing Clin. Electrophysiol..

[92] Fenici R., Brisinda D., Meloni A.M. (2005). Clinical application of magnetocardiography. Expert Rev. Mol. Diagn..

[93] Dekie L., Kleiman R.B. (2022). False Negative ECG Device Results May Increase the Risk of Adverse Events in Clinical Oncology Trials. Ther. Innov. Regul. Sci..

[94] Sakib S., Fouda M.M., Al-Mahdawi M., Mohsen A., Oogane M., Ando Y., Fadlullah Z.M. Noise-Removal from Spectrally-Similar Signals Using Reservoir Computing for MCG Monitoring. Proceedings of the ICC 2021—IEEE International Conference on Communications.

[95] Mohsen A., Al-Mahdawi M., Fouda M.M., Oogane M., Ando Y., Fadlullah Z.M. AI Aided Noise Processing of Spintronic Based IoT Sensor for Magnetocardiography Application. Proceedings of the ICC 2020—2020 IEEE International Conference on Communications (ICC).

